# Current Status and Perspective on the Use of Viral-Based Vectors in Eukaryotic Microalgae

**DOI:** 10.3390/md20070434

**Published:** 2022-06-29

**Authors:** Omayra C. Bolaños-Martínez, Ganesan Mahendran, Sergio Rosales-Mendoza, Sornkanok Vimolmangkang

**Affiliations:** 1 Department of Pharmacognosy and Pharmaceutical Botany, Faculty of Pharmaceutical Sciences, Chulalongkorn University, Bangkok 10330, Thailand; omayra.cbm@hotmail.com (O.C.B.-M.); mahendran0007@gmail.com (G.M.); 2 Center of Excellence in Plant-Produced Pharmaceuticals, Chulalongkorn University, Bangkok 10330, Thailand; 3 Laboratorio de Biofarmacéuticos Recombinantes, Facultad de Ciencias Químicas, Universidad Autónoma de San Luis Potosí, Av. Dr. Manuel Nava 6, San Luis Potosí 78210, Mexico; rosales.s@uaslp.mx; 4 Sección de Biotecnología, Centro de Investigación en Ciencias de la Salud y Biomedicina, Universidad Autónoma de San Luis Potosí, Av. Sierra Leona 550, Lomas 2a Sección, San Luis Potosí 78210, Mexico

**Keywords:** biopharmaceuticals, recombinant proteins, transient expression, viral vectors

## Abstract

During the last two decades, microalgae have attracted increasing interest, both commercially and scientifically. Commercial potential involves utilizing valuable natural compounds, including carotenoids, polysaccharides, and polyunsaturated fatty acids, which are widely applicable in food, biofuel, and pharmaceutical industries. Conversely, scientific potential focuses on bioreactors for producing recombinant proteins and developing viable technologies to significantly increase the yield and harvest periods. Here, viral-based vectors and transient expression strategies have significantly contributed to improving plant biotechnology. We present an updated outlook covering microalgal biotechnology for pharmaceutical application, transformation techniques for generating recombinant proteins, and genetic engineering tactics for viral-based vector construction. Challenges in industrial application are also discussed.

## 1. Introduction

Microalgae are unicellular microorganisms found in marine and freshwater ecosystems over a wide range, from very small (a few micron) to large (a few hundreds of microns). They can rapidly produce biomass from solar energy, CO_2_, and nutrients, such as nitrogen, sulfur, and phosphorous. Simple maintenance and cultivation in artificial environments offer a profitable platform to produce and extract bioactive compounds compared with other bioresources. Here, microalgae produce various metabolites with applications in pharmaceutical, cosmetic, bioenergy, and food/feed industries [1,2]. Various microalgae-derived products for food and feed have already been commercialized by different companies worldwide, including A4F-Algae 4 Future (Portugal), Blue Biotech (Germany), DIC Lifetec (Japan), E.I.D Parry (India), Ocean Nutrition (Canada), Phycom (Netherlands), Chlorella Co. (Taiwan), and Solazyme, Inc. (San Francisco), all of which used their bioactive compounds as colorants, additives, or supplements [3].

Biopharmaceuticals are complex molecules of biological origin used to diagnose, prevent, treat, and cure diseases or conditions in human beings and animals. According to their biological structure, biopharmaceuticals can be classified into amino acids, nucleic acids, and vaccines. In biopharmaceutical terms, these molecules are specifically produced under biotechnological processes based on genetically engineered organisms used as an expression host [4]. The main organisms used here are bacteria, yeast, mammalian cells, and insect cells, with each system having their own advantages as well as limitations [5,6,7,8].

Recently, microalgae have attracted increasing scientific interest due to their versatile growth and functional metabolic properties, as well as their biopharmaceutical production. Microalgae possess distinct attributes that have attracted the attention of biotechnologists, who have developed advanced genetic and molecular tools to leverage microalgae as green bioreactors to produce biopharmaceuticals. These attributes include their ability to grow and culture under heterotrophic, autotrophic, and mixotrophic conditions, the capacity to realize post-translational modifications and proper protein maturation, and the distinction of some microalgae species as “Generally Recognized as Safe” by the Food and Drug Administration (FDA). This status is conferred to any substance, chemical, or a whole organism that is safe for human consumption, owing to the absence of pathogens, microorganism, or related endotoxins. Mostly heterotrophic microalgae are FDA-approved for biotechnological applications due to their large-scale growing capacity and high cell density compared with other organisms [9,10]

**Figure 1:**
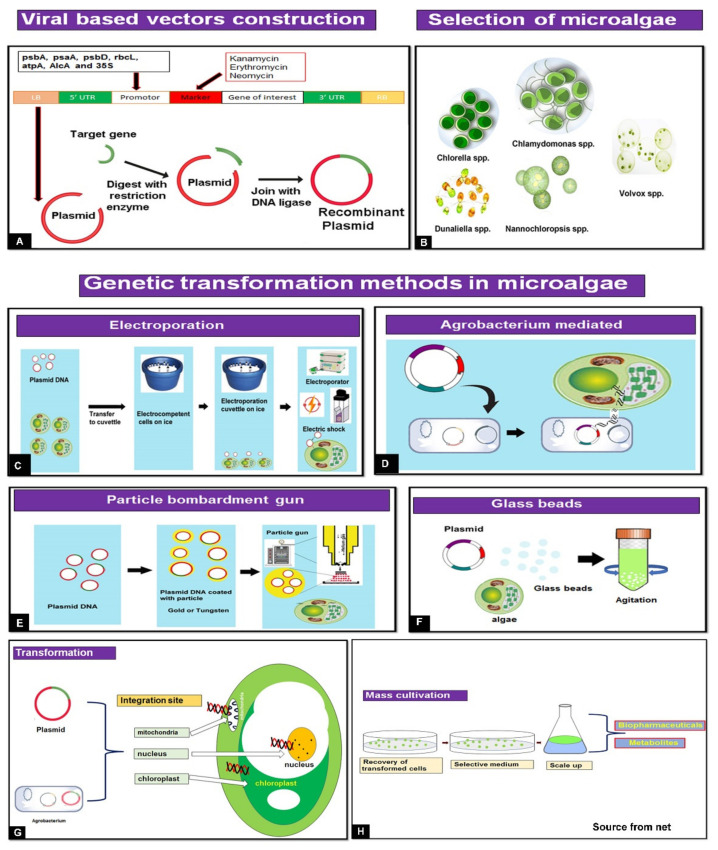
Overview of microalgal biotechnology for biopharmaceutical application. The essential components are from the vector design and selection of gene interests, microalgal hosts, and methods of transformation to finally obtain either bioactive metabolites or biopharmaceuticals. (**A**). Plasmid construction and transfer to *Agrobacterium*. (**B**). Selection of microalgae for genetic transformation. (**C**). Method to transfer plasmid DNA using electroporation. (**D**). Introduction to target gene through the *Agrobacterium*-mediated method. (**E**). Stepwise protocol for the transfer of genes. (**F**). Traditional algae transformation method (glass beads). (**G**). Transformation methods (direct or Agrobacterium mediated) and integration into algae cell. (**H**). Stages of development for large-scale production of valuable biopharmaceuticals.

To increase the yield and accelerate time to obtain and improve biopharmaceutical quality, microalgae biotechnology uses various expression methods and genetic and molecular biology strategies. These methods include stable nuclear and chloroplast expression and, in recent years, transient expression using viral-based vectors that allow high protein accumulation in a short period of time. However, the method using *Agrobacterium tumefaciens* transformation makes oral formulations of algal biomass unusable due to residual bacteria. Conversely, viral vectors for this purpose are limited and are mainly designed using elements derived from plant viruses. We present an updated outlook covering microalgal biotechnology for pharmaceutical applications, transformation techniques for obtaining recombinant proteins, and genetic engineering tactics for viral-based vectors construction (Figure 1). Finally, we discuss the potential challenges in industrial application.

## 2. Genetic Engineering Transformation Methods for Biopharmaceutical Production of Microalgae

During the last 20 years, 40 different microalgae species genetic engineering methods have been developed [11,12]. *Chlamydomonas reinhardtii*, *Dunaliella salina*, *Volvox carteri*, *Haematococcus pluvialis*, and *Phaeodactylum tricornutum* are widely used microalgae for transforming foreign transgene expression studies, as well as biopharmaceutical protein production [13,14,15,16,17]. Here, microalgae genomes, such as nuclear, chloroplast, and mitochondrial transformation protocols, have been explored. In microalgae, four traditional methods are widely used to deliver foreign genes into microalgal genomes, including agitation with glass beads [18], particle bombardment [19], electroporation [20], and *Agrobacterium*-mediated transformation [21,22,23,24,25]. Of these methods, glass beads and *Agrobacterium* do not require any specialized apparatus, are less labor-intensive, and are relatively fast [18,26]. Additionally, bacterial conjugation as well as natural and liposome-mediated transformation also have been employed, each of these exhibiting their own advantages and drawbacks. The most notable disadvantages presented for some methods include the need for optimizing the transformation conditions, the low efficiency, and the high cost of the equipment or interface used [27].

Using the agitation method, transformation involves agitating wall-deficient cells/protoplasts of microalgae with foreign genes, glass beads, and polyethylene glycol (surfactant) [28,29,30]. This method can be used for both nuclear and chloroplast transformation. Furthermore, studies show cell wall-removed protoplasts are sufficient for gene transformation in *Chlorella ellipsoidea* [31]. Glass bead agitation has also been reported in chloroplast genetic engineering in *C. reinhardtii* using agitation of DNA/cell suspensions with glass beads [32]. The glass bead method also includes low transformation efficiencies due to thick cell walls, agitation duration, velocity, and surfactant concentration [12,33,34,35]. Table 1 presents and compares the limitations of different transformation methods.

The *Agrobacterium*-based transformation method has previously been applied to *C. reinhardtii* [23,50,58], *H. pluvialis* [22,59], *Chlorella vulgaris* [60], *Parachlorella kessleri* [61], *Dunaliella bardawil* [46,47], *D. salina* [62], *Euglena gracilis* [63], *Cenedesmus almeriensis* [64], and *Dictyosphaerium pulchellum* [65]. According to Bashir et al. (2018), efficiency transformation using the Agrobacterium-based method was 50-fold higher that the glass bead method. However, different transformation efficiencies have been reported with *Agrobacterium*-based protocols [23,50,58]. Factors such as co-cultivation temperature, optical density, infection time, pre-culture duration, and acetosyringone concentration can substantially affect transformation efficiency [47,60]. In a study by Kumar et al. (2004), the *Agrobacterium*-based method performed equally as well as electroporation for stable integration into *Parachlorella kessleri* [61].

Electroporation is the most common and effective method for performing high-intensity electric pulses across the microalgae cell membranes to allow exogenous DNA to pass through cells [66,67,68]. This method has been reported in *C. reinhardtii* [20,38,39], *Nannochloropsis limnetica* [40], *D. salina* [41], *Scenedesmus obliquus* [42], *Monoraphidium neglectum* [43], *Chlorella pyrenoidosa* [44], *C. vulgaris* [69], *Chlorella zofingiensis* [70], and *Nannochloropsis oculata* [45]. Advantages include a rapid protocol, low labor, and high speed. Electroporation has been also been reported with transformation efficiencies up to 100-fold over agitation [12]. However, transformation efficiencies may be affected by electric-strength, pulse, and cell wall complexity [20,71,72].

Particle bombardment is an early and highly reproducible transformation method due to its ability to deliver genes into the nucleus, mitochondria, and chloroplast genomes without disturbing the cell walls [19,49,73,74]. This method is based on a DNA-coated ejection device with tungsten or gold metal particles that can detect target cells. Successful transformation using particle bombardment have previously been reported for *C. reinhardtii* [9,49,50,51,75,76], *D. salina* [77], *Haematoccucs pluvialis* [59], *V. carteri* [48], *P. tricornutum* [52], *Cyclotella cryptica* and *Navicula saprophila* [53], *Cylindrotheca fusiformis* [54], and *Schizochytrium* sp. ATCC 20888 [78,79].

Among these techniques, the particle gun method is the most efficient for direct DNA delivery into cells. Generally, the gene gun method shows high transformation efficiency; however, this method is costly. Both particle bombardment and electroporation can be applied to transfer not only endogenous DNA but also proteins into microalgae cells. The most important application introduced Cas9 protein-gRNA ribonucleoproteins (RNPs) into microalgae, namely, into *C. reinhardtii*, *P. tricornutum*, and *Tetraselmis* sp. cells, for DNA-free genome editing [80,81,82,83].

In addition to the aforementioned methods used to introduce foreign DNA into microalgae cells, other transformation methods are also available. Hawkins and Nakamura (1999) showed *Chlorella* sp. protoplast cells and plasmids can be generated by mixing with polyethylene glycol and dimethyl sulfoxide for human growth hormone gene transformation [84]. Similarly, Liu et al. (2013) described novel, simple, reliable, and cost-effective transformation of *C. ellipsoidea* protoplast cells by mixing foreign DNA with PNC solution (NaCl, CaCl_2_, and 40% PEG 4000) [71]. Other methods include stable nuclear transformation systems for *Pleurochrysis carterae* using polyethylene glycol (PEG)-mediated transfer of hygromycin B-resistance genes [85]. Recent reports present genetic transformation of microalgae by bacterial conjugation [86,87] and gene injection [88]. In addition to these techniques, other emerging methods, such as cell-penetrating peptides, nanoparticles, metal–organic frameworks, and liposomes, have not yet been demonstrated in microalgae [12,89,90,91].

## 3. Microalgae Nuclear and Chloroplast-Based Expression

Microalgae contain nuclear, mitochondrial, and chloroplast genomes, each of which have their own transcription, translation, and post-translation properties [92]. Nuclear expression in microalgae offers numerous benefits, such as targeting recombinant protein expression in specific organelles, protein glycosylation, post-translational modification, and secretion [93]. In nuclear-based expression, the position of an exogenous gene into a microalgal genome occurs as a random insertion and usually transgenic cells are selected via phenotypic variation or antibiotic resistance. Generally, this approach results in low yields. Although the reasons for this phenomenon are not completely understood, possible explanations could be attributed to the RNA-silencing process, transcript instability, positional effects of transgenes, and an inaccessible chromatin structure [94].

Using chloroplasts to express foreign genes has become a promising alternative to the nuclear genome. Microalgae chloroplasts serve as the main cell factory for synthesizing several metabolic pathway enzymes and appropriate transformation objects for producing isoprenoids, carbohydrates, lipid, carotenoids, pigments, fatty acids, and proteins [95,96]. Further, this organelle lacks a gene-silencing mechanism and may be used to protect proteins from degradation and involve some post-translation modifications, such as phosphorylation. These multiple functions in a single cell organelle are the most important traits for its heterologous gene expression in microalgae [97,98]. For delivery, the foreign gene must pass through several membranes, which represent a greater challenge. The preferred method to achieve this goal is particle bombardment. In particular, *C. reinhardtii* has been descried in numerous transformation studies for producing foreign proteins due to the chloroplast genome being fully sequenced and offering a unique advantage in the transformation system [99]. Further, various transformation methods have been reported for *C. reinhardtii* chloroplasts, among which are the marker-free chloroplast transformation system [100] and glass bead agitation using cell wall-deficient cells [28,29,30]. Finally, a chloroplast transformation system based on electroporation has also been developed for *Phaeodactylum tricornutum* [101].

## 4. Algal Biotechnology in Pharmaceutical Applications

In biochemistry, metabolites are defined as small molecules of <1.5 kilodaltons (KDa) that act as intermediates or end products in cellular metabolism and are classified as primary and secondary. Primary metabolites are directly involved in growth, development, and reproduction, whereas secondary are not implicated in these processes but offer an important ecological function and are typically linked to specific environmental conditions or developmental stages [102]. In microalgae, diverse bioactive metabolites have been studied for their antifungal, anticancer, antibacterial, and immunosuppressive properties [103,104,105,106,107].

Further, bioactive compounds obtained from microalgae, such as β-carotene, polyunsaturated fatty acids (Omega-3), clionasterol, phycocyanin, lutein, astaxanthin, canthaxanthin, fucoxanthin, zeaxanthin, docosahexaenoic acid (DHA), and eicosapentaenoic acid (EPA), can be applied as nutraceuticals, food additives, or in the cosmetics industry. Amino acids, such as tryptophan, lysine, leucine and arginine, vitamins B and E, essential minerals, and carbohydrates, are used in human and animal nutrition. Further, metabolites obtained from microalgae can be used in biofertilizer production as a source of nitrogen- and phosphorous-rich biomass residues as feedstock and in the bioenergy industry as bulk oil and biomass residue feedstock for jet fuel, biodiesel, bioethanol, biogas, biochar, and biohydrogen production. Furthermore, some microalgae strains can be used in wastewater treatment by reducing the amount of nitrogen, phosphate, and chemical oxygen demand, as well as removing heavy metals (copper (Cu), iron (Fe), manganese (Mn), and zinc (Zn)) and pharmaceutical pollutants (triclosan and hormones (17β-estradiol and 17α-ethinylestradiol) [108,109,110,111,112,113]. Interestingly, potential industrial applications and commercialization of microalgae-derived biomass and bioactive compounds in the food industry has recently been explored by Camacho et al. (2019). This analysis introduced the potential for formulation as prebiotics or as part of functional food/feed for human and animal consumption. Further, various industries can commercialize products, including phycocyanin, lutein, β-carotene, astaxanthin, eicosapentaenoic acid (EPA), and docosahexaenoic acid (DHA) (ω–3), derived from microalgae to be used as food colorants or supplements [3]. Currently, different species of microalgae have been used in the food/feed industry, such as *Porphyridium cruentum*, *Pavlova salina*, *Tisochrysis lutea*, *Chaetoceros muelleri*, *Nannochloropsis* spp., *Skeletonema* spp., *Thalassiosira pseudonana*, *Schizochytrium* sp., and *Crypthecodinium cohnii*, and wastewater bioremediation, including *Scenedesmus obliquus*, *Franceia* sp., *Ankistrodesmus* sp., *Tetraedron* sp., *Chlorella* sp., and *Mesotaenium* sp. [114].

Conversely, using single-cell engineering microalgae as a green factory to produce biopharmaceuticals includes recombinant expression of numerous antigenic proteins that act as human and animal vaccine candidates against viral or bacterial diseases and parasitic infections. Among these candidates, expression of viral epitopes from Zika virus [115], avian influenza [116], human papillomavirus [117], hepatitis B [41], and human immunodeficiency virus (HIV) [118], as well bacterial proteins from *Staphylococcus aureus* [76] and *Histophilus somni* [119], are well studied. Regarding parasitic infections, proteins from *Plasmodium falciparum* that cause malaria are also expressed in microalgae [120,121]. Furthermore, microalgae are also used to produce monoclonal antibodies, hormones, cytokines, growth factors, immunotoxins, and proteins to prevent non-communicable diseases [122,123,124,125]. A detailed recompilation of biopharmaceuticals produced in microalgae are summarized in Table 2. In addition, these recombinant microalgae cells can be utilized as an effective oral drug delivery platform formulated as pills, tablets, or freeze-dried cells [9]. A study by Kwon et al. (2019) demonstrated that the green fluorescent protein (GFP) expressed in chloroplasts of *C. reinhardtii* remained intact after biomass lyophilization [126].

## 5. Viral-Based Expression Vectors for Recombinant Protein, Vaccine, and Biopharmaceutical Production

Currently, biotechnology and genetic engineering is harnessing numerous viruses or their component parts to produce heterologous proteins for human and animal use. Given the expression of epitopes from influenza A virus can be fused with the hepatitis B core antigen in *Nicotiana benthamiana* plants, generation of virus-like particles (VLPs) in insect cells for the human papilloma virus as a vaccine-delivery vehicle for genetic material can generate an immune response in the human body, as recently developed for a COVID-19 vaccine [160,161,162]. Furthermore, polymerases and reverse-transcriptases from viral origins, in addition to elements such as transcriptional promoters, terminators, silencing suppressors, and internal ribosomal entry sites, form part of a molecular toolbox for genetic engineers, biologists, and biotechnologists.

The common approach for generating viral-based expression vectors involves inserting a determinate viral genome sequence into an expression vector downstream of a cell-type-specific promoter. The coding sequence of a heterologous gene is then inserted into the viral genome sequence as part of a viral polyprotein or downstream to a subgenomic promoter. The construct is then transferred to host cells for transcription and subsequent translation processes by host molecular machinery [163]. During the last decade, the design, generation, and use of viral-based expression vectors for producing heterologous proteins have gained increasing scientific interest, mainly in the plant biotechnology field. To achieve this goal, expression strategies have focused on RNA and DNA plant viruses, of which tobamovirus, comovirus, potexvirus, and geminivirus are the most exploited genera.

Developing and applying this approach has followed an interesting path. First, by creating first-generation expression vectors or full virus strategies based on expression of the gene of interest (GO), this approach has also produced its own viral genes and subsequent translation as an individual antigenic or fusion protein on the C-terminal of the capsid protein (CP). Using these vectors, several immunogens have been produced, reaching up to 10% of the total soluble protein (TSP) in *Nicotiana benthamiana* plants. However, stability is negatively related to insert size, hence the proteins larger than 30 KDa are poorly expressed in a chimeric CP form and epitopes should be 25 amino acids at maximum length [164,165,166]. These drawbacks slowed the development of second-generation viral vectors, whereby using a full virus was replaced with a deconstructed virus genome containing essential elements for replication and non-viral sequence integration to accomplish other functions, such as replicon formation using T-DNA delivered via *A. tumefaciens*. Using *Agrobacterium* for DNA delivery offers considerable advantages given the efficient transfer capacity by infiltration of plant leaves. Plants species using this approach include spinach, sunflower, red beetroot, and *N. benthamiana,* presenting maximum yields up to 50% of TSP in a 4–5 day period where the size of the GO can be up to 2 Kb and proteins of 80 KDa can be produced [167,168,169].

Special attention should be directed toward DNA virus-based vectors, specifically those applying elements from geminivirus, a twinned icosahedral virus with a single-strand DNA (ssDNA) arranged in one (monopartite) or two components (bipartite) encoding proteins essential for the replication process, pathogenicity, suppression of plant gene silencing, and intercellular and long-distance movement of the virus [170,171]. In general, these vectors are based on a transient expression system, the advantages of which include rapid product expression, high production rate, flexibility, and scalability. A geminivirus engineered for biopharmaceuticals is *Bean Yellow Dwarf Virus* (BeYDV), which has been modified to leverage its Rep protein under independent promoter control. With this strategy, diverse BeYDV-based expression vectors have been engineered and an assortment of antigens and monoclonal antibodies have been generated [172,173,174]. For microalgae, the geminiviral vector pBYR2e was used for expression of the receptor-binding domain (RBD) from SARS-CoV-2 and fibroblast growth factor (bFGF) in two freshwater microalgal species. Yields reached up to 1.61 μg/g and 1.14 μg/g for RBD when expressed in *C. reinhardtii* and *C. vulgaris*, respectively [35]. Conversely, Berndt et al. (2021) reported expression of RBD-fused GFP in *C. reinhardtii.* Interestingly, the protein targeted three different cellular localizations: (i) in the endoplasmic reticulum–Golgi pathway; (ii) secreted out of the cell into the culture media; and (iii) directed to the chloroplasts. In the latter, although under higher expression, the protein appeared to be truncated by ~5 kDa at the amine end, whereas the end targeted to the ER was produced with the expected size and correct amino acid sequence. For obtaining proteins, the transgene was placed into the pBR9 and pOpt vectors; in particular, the pBR9 vector containing the *sh ble* zeocin resistance selection marker with a food and mouth disease virus (FMDV) 2A self-cleaving sequence placed between the coding sequences, resulted in accumulation of two separate proteins [175].

Another geminivirus-based vector, named Algevir, has been developed with diverse antigenic proteins and epitopes expressed in the marine microalgae *Schizochytrium* sp., which was engineered using the Rep protein and origin of replication (Ori) from the begomovirus *Ageratum enation* to produce and replicate circular DNA carrying the GO and AlcR gene, as well as the AlcA promoter from *Aspergillus nidulans* to obtain ethanol-induced expression. This innovative system has produced viral and bacterial proteins at a maximum level production of 1.25 mg/g fresh biomass for GP1 from *Zaire ebolavirus* [79]. Table 3 shows the viral-based vectors used for biopharmaceutical production in microalgae. However, yields produced in microalgae with a nuclear approach and using viral-based vectors do not fully outcompete those produced in chloroplasts whereby targets allow production of 3.28 mg/L of culture medium [176]. The strategy based on protein production in this organelle requires a long time and construction of detailed vectors containing specific sequences for integration by homologous recombination. Here, optimizing viral-based vectors is needed to increase the protein yield and improve stability, which requires transient expression as a primary approach given that some transgene products may become toxic for host cells, leading to very low yields under stably transformed lines. Alternatively, microalgae viruses can be naturally used to drive gene expression at different infection stages and viral elements can be explored throughout the design process of novel viral-based vectors or when improving current models. Updating the functions of viral genes and the genome composition is an important requirement for executing a rational design in which regulatory elements, such as promoters, terminators, or replication proteins, help reach strong GO expression. Finally, exploring the possibility of directly purifying recombinant proteins using elements from lytic viruses presents an alternative approach [177].

## 6. Design of a Viral-Based Vector for Microalgae Use

In the virosphere, many species are capable of infecting microalgae. In addition to triggering high mortality rates, such species can reprogram host metabolism, including photosynthesis and important cycling processes, such as central carbon metabolism, phosphorus, nitrogen, and sulfur [178].

To date, a total 63 virus that infect eukaryotic microalgae have been isolated and cultured in the laboratory, whereby 50.79% contain dsDNA as genomic material, 15.8% ssDNA, 1.58% dsRNA, and 22.2% ssRNA, whereas 7.93% have not yet been classified [163,179]. Recently, a list of 10 isolated and characterized viruses was published by Sandaa et al. (2022) [180]. These viruses can infect marine haptophytes species. Here, a rational design of a microalgal-specific viral vector to achieve higher protein yields, using viral elements that naturally infect microalgae, could be a promising strategy.

In a study published by Kadono et al. (2015) [181], a set of five potential promoter regions located upstream of the replication-associated protein (VP3) or structural protein (VP2), coding genes for three marine diatom-infecting viruses (DIVs), were evaluated and compared in the Pennales diatom *Phaeodactylum tricornutum* as a heterologous host (Table 4). The gene-encoding fucoxanthin chlorophyll a/c-binding protein (fcp) was used as an endogenous promoter and eGFP as a protein reporter. In addition, the extrinsic promoter, such as *Cauliflower mosaic virus* 35S (CaMV35S), cytomegalovirus (CMV), and nopaline synthase gene (nos) promoter, were also used. The results show the novel promoter ClP1 mediated significantly higher transcription and translation rates according to mRNA transcripts and flow cytometry analysis, respectively. Further, the abundance of eGFP mRNA transcripts in the stationary phase were higher than those found in the log phase under both low and standard nutrient culture conditions.

In addition to DIVs, other viruses can help explore their genetic elements and design a novel viral-based vector, particularly those with ssDNA or dsRNA genomes. Among them, viral species infecting the most commonly studied microalgal, such as the genus *Chlorella*, may offer a useful genetic toolbox. For example, the *Paramecium bursaria Chlorella virus 1* (PBCV-1), a large dsDNA virus (>300 kb) infecting the green microalgae *Chlorella variabilis* NC64A, is now a model system for studying DNA virus/algal interactions, which has also been tested for biomass saccharification with subsequent bioethanol production and proteins involved in cell wall degradation [182,183,184,185]. Another virus fully sequenced that infects the *Chlorella* genus with potential biotechnology application are those that exclusively multiply in Syngen 2–3 or SAG 3.83 cells, which could lead to specific protein expression in microalgae strains. The prototype viruses are only Syngen viruses—NE5 (OSy-NE5) and *Acanthocystis turfacea* chlorella virus (ATCV-1) [186,187].

## 7. Challenges and Perspectives

In recent years, the current pandemic has pushed progress of several biomedical technologies, e.g., RNA vaccines and adenovirus-based vaccines. Based on these advances, what are the key insights from the field of algae-based biopharmaceuticals? Biopharmaceuticals using algae are considered a promising alternative for improving global health. Algae offer low production costs and some species are already used at industrial levels in the food industry and thus are considered safe for use as delivery vehicles, especially oral formulations. However, although the proof of concept for using algae to produce and even deliver biopharmaceutical has been reported by several groups, a number of challenges remain to be addressed in this field, including improving recombinant protein yield productivity.

Another critical path that deserves research attention in developing algae-made biopharmaceuticals is related to regulation. Defining the main guidelines for specific regulations applied to this type of biological agent is a major priority task. Performing clinical trials requires translating prototypes generated in academic labs to facilities with good laboratory practices that can approve and perform clinical trials. Moreover, implementing GMP-compliant processes in cooperation with pharmaceutical companies is urgently needed.

The current pandemic has increased support from several countries to invest in biomedical research and strengthen the developmental path for drugs and biologics. For example, several developing and emerging countries are increasing funding for research on innovative platforms for biopharmaceuticals production, including Thailand and México. We consider that the innovative green platforms required to produce biopharmaceuticals are a promising niche that could be accelerated by such initiatives. However, this should be a mid-term goal considering that conventional production systems with well-established regulatory frameworks will be the priority for such countries to provide rapid solutions for immediate needs. As biopharmaceuticals are inherently more complex than conventional chemical drugs, they demand a more complicated manufacturing process with varying quality and demands for extensive processes and product understanding. In addition, downstream processing represents another bottleneck. For algae, eliminating large amounts of lipids present in total extracts should be studied and the impact of differential glycosylation compared with mammalian glycosylation is another aspect that deserves attention.

Although the good manufacturing practice (GMP) standards of various regulatory authorities and international organizations are very similar and appropriate in addressing the manufacturing challenges, introducing innovative platforms always presents challenges. This challenge is exacerbated in developing or emerging countries that require affordable biopharmaceuticals. For instance, a recent study by Rahalkar et al. (2021) revealed that, in several emerging countries, the lack of standardized biosimilar development criteria and regulatory convergence across agencies led to challenges in multi-country biosimilar development, limiting our ability to introduce new, cheaper biosimilars into the market [188]. Unfortunately, for biopharmaceuticals produced in algae, this remains an ongoing challenge.

Although using viral vectors improves efficiency in expression systems, using *Agrobacterium* presents the need for complex purification steps to eliminate bacterial endotoxins. Therefore, expanding stable transformation systems to express viral replicons under an inducible approach is a possible solution to this limitation. Avoiding antibiotic-resistant markers is another challenge when designing vectors. Alternative markers, such as nutrient-selective markers, are accruing more interest. Another possibility is developing oral formulations subjected to less strict regulations. It is clear that this field is still in its infancy; thus, exploring new constructs optimized for model species, especially *C. reinhardtii*, are required. Special emphasis on developing vectors based on new algae viruses is crucial.

## 8. Concluding Remarks

Although using viral-based expression systems in algae is still new, this technology has immense potential to revolutionize the algae-based biopharmaceuticals field by offering higher yields and shorter production times compared with chloroplast and nuclear stable transformation methods. The following decade will be critical, as technology will benefit from refreshed interest when supporting biomedical research in response to the COVID-19 pandemic. Research and development goals should be focused not only on generating prototypes in academic labs but also on critical regulatory issues to ensure the success of new products that enter the market and ultimately benefit human health, especially in developing and emerging countries. On February 2022, Medicago, a Canadian company, and GlaxoSmithKline (GSK) announced approval by the Health agency in Canada of COVIFENZ^®^, a COVID-19 vaccine produced in plants. This is a milestone, as it is the first vaccine produced using a green platform approved for human use. Will algae-based products reach the same success? The following decade will be crucial in addressing this goal.

## Figures and Tables

**Table 1:** Comparison and limitations of genetic transformation methods in microalgae.

Method	Advantage	Disadvantage	Integration Site	Transformation Efficiency	Microalgae Species	Reference
Glass beads	Simple, controlability, high cell-survival rate, affordable, and minimal physical damage to cells	Cell wall removal and low transformation efficiency	Nucleus	~10^3^ µg DNA^−1^	*Chlamydomonas reinhardtii*	[18]
				NR	*Dunaliella salina*	[36]
				NR	*Platymonas subcordiformis*	[37]
Electroporation	Simple, affordable equipment, and high transformation efficiency	Transformation frequency affected by higher pulse strength and length, medium composition, temperature and membrane characteristics	Nucleus	6 × 10^3^ per µg of DNA	*Chlamydomonas reinhardtii*	[38]
				2.5 × 10^4^ per µg of DNA		[39]
				1.1 × 10^7^ per µg of DNA	*Nannochloropsis limnetica*	[40]
				NR	*Chlamydomonas reinhardtii*	[20]
				NR	*Dunaliella salina*	[41]
				NR	*Scenedesmus obliquus*	[42]
				NR	*Monoraphidium neglectum*	[43]
				NR	*Chlorella pyrenoidosa*	[44]
				NR	*Nannochloropsis oculata*	[45]
*Agrobacterium-*mediated	Transformation of large DNA fragments, simple, stable, and efficient	Variation of transformation efficiencies and transformation frequency affected by physical and chemical factors, silenced transformants, lower number of multiple insertions	Nucleus	311–355 × 10^−6^	*Chlamydomonas reinhardtii*	[23]
				NR	*Haematococcus pluvialis*	[22]
				41.0 ± 4 CFU per 10^6^ cells	*Dunaliella bardawil*	[46,47]
Biobalistic	Most effective method for the transformation of chloroplasts/nuclear, multiple copies insertion. More DNA integration and copy number	Cost effective, required specialized equipment, and recovery low	Nuclear/Chloroplast Genome	~2.5 × 10^−5^ DNA	*Volvox carteri*	[48]
				1.9 × 10^−6^ to 4.2 × 10^−6^ per µg of DNA 10^8^	*Chlamydomonas reinhardtii*	[49,50]
				NR		[51]
				NR	*Phaeodactylum tricornutum*	[52]
				NR	*Cyclotella cryptica* and *Navicula saprophila*	[53]
				NR	*Cylindrotheca fusiformis*	[54]
Silicon carbide whiskers	Similar protocol	Low transformation frequency	Nucleus	NR	*Chlamydomonas reinhardtii*	[55,56]
Lithium acetate/polyethylene	Simple operation, low cost, less damage to the host cells and high transformation efficiency	Growth rate transformation temperature and plasmid concentration	Nucleus	113 colonies μg^−1^ DNA	*Dunaliella salina*	[57]

NR: Not Reported.

**Table 2:** Production of recombinant biopharmaceuticals proteins in microalgae.

Microalgae Strain	Transformation Method	Integration Site	Protein Expressed	Yields Obtained	Application	Reference
*Dunaliella salina*	Lithium acetate/PEG	Nucleus	SKTI	0.68% TSP	Antivirus and anticancer	[57]
	*Agrobacterium*-mediated transformation		H5HA	225 µg TSP/2g	Avian influenza	[127]
	Electroporation		HBsAg	3.11 ± 0.50 ng/mg	Hepatitis B	[41]
	Glass beads		VP28	3.04 ± 0.26 ng/mg and 78 µg/100 mL culture	White spot syndrome in crayfish	[128]
	Biolistics	Chloroplast	sTRAIL	0.67% TSP	Tumor cells and virus-infected cells	[129]
*Chlamydomonas reinhardtii*	*Agrobacterium*-mediated transformation	Nucleus	HBcAgII	0.05% TSP	Hypertension	[130]
			IFN-α2a	NA	Immunity	[131]
			RBD	1.61 µg/g FWB	COVID-19	[35]
			bFGF	1.025 ng/g FWB	Growth factor	
	Glass beads	Chloroplast	HPV16 E7 mutated form r E7GGG-His6, E7GGG and E7GGG-FLAG	E7GGG-His6 (0.02%), E7GGG (0.1%) and E7GGG-FLAG (0.12%) TSP	Cancer	[117]
			WSSV VP28	NA	White spot disease in shrimp	[132]
			hGH	0.5 mg hGH/L	Growth Hormone	[32]
			dsRNA	NA	Yellow head virus infection in shrimp	[133]
	Biolistics		ctxB-pfs25	0.09% TSP and 20 µg/mL	Malaria	[51]
			pfs25 and pfs28	Pfs25 (0.5%) and Pfs28 (0.2%) TSP		[134]
			c.r.pfs48/45	NR		[75]
	Glass beads	Nucleus	AMA1/MSP1-GBSS	0.2 to 1.0 mg of protein/mg		[120]
			hVEGF-165, hPDGF-B, and hSDF-1	0.06% TSP, 0.003% TSP, 0.0006% TSP	Tissue hypoxia, wound healing	[135]
			*P24,* CpP24, CrP24, P24w	0.25% TSP	AISD	[118]
			hEGF	0.2%–0.25% TSP (40 mg/L)	hEGF deficiency	[136]
			Endolysin (Cpl-1 and Pal)	~1.3 mg/g ADW	*Streptococcus pneumoniae* infection	[137]
			ALFPm3	0.35% TSP	Anti-bacteria, anticancer, and antiviral activity	[138]
			IF	NA	Autoimmune disease pernicious anemia	[139]
	Biolistics	Chloroplast	αCD22	0.7% TSP	Cancer	[124]
			83K7C	100 mg/1 g of DAB	Anthrax	[140]
			HSV8 scfv	0.5% TSP	Herpes simplex virus	[141]
			HSV8-lsc	>1% TSP	Herpes simplex virus	[142]
			M-SAA	0.25% TSP	Protection against intestinal bacterial and viral infections in newborns	[143]
			apcA and apcB	2–3% TSP	Inhibit the S-180 carcinoma in mice	[144]
			hMT-2	NA	UV-B effects	[145]
			CTB:p210	60 µg/g of FWB	Atherosclerosis	[146]
			Ara h 1 and Ara h 2	NA	Peanut allergy	[147]
			Bet v 1.0101	0.01 and 0.04% TSP	Allergy	[148]
			IL-2 and PfCelTOS	1.5% TSP	Malaria	[121]
			IFN-β1	NA	Multiple sclerosis	[149]
			VEGF	0.1% TSP	Depression and pulmonary arteries	[149]
			HMGB1	1% TSP	Response of the brain to neural injury and wound healing	[149]
			CelK1	0.003% TSP	Bioethanol and biogas production	[150]
	Biolistics	Nucleus	huBuChE	0.4% TSP	Pesticide poisonings	[151]
	Electroporation	Nucleus	Mytichitin-A	0.28% TSP	Growth inhibition of fungi, viruses, parasites, and bacteria	[152]
			ToAMP4	0.32% TSP	Antimicrobial	[153]
			hLF	1.82% TSP	Antibacterial	[154]
*Schizochytrium* sp	*Agrobacterium*-mediated transformation	Nucleus	HER-2, MUC1, MAM-A, and WT1	637 µg/g FWB	Breast cancer	[155]
			ZK1, ZK2, ZK3, and LTB	365 µg/g FWB	Zika disease	[115]
			LTB:RAGE	380 μg/g FWB	Alzheimer disease	[156]
			GP1 and LTB	1.25 mg/g FWB (6 mg/L of culture)	Ebola	[79]
*Schizochytrium* sp. ATCC 20888	Biobalistic	Nucleus	HA	5–20 mg/l	Influenza	[78]
*Chlorella vulgaris*	*Agrobacterium*-mediated transformation	Nucleus	RBD	1.14 µg/g FWB	COVID-19	[35]
			bFGF	1.61 ng/g FWB	Growth factor	
*Chlorella* sp	Electroporation	Nucleus	Scygonadin and hepcidin	NA	Antibacterial	[157]
*Chlorella sorokiniana* ATCC-22521) or *Chlorella vulgaris* C-27	PEG	Nucleus	hGH	200–600 ng/mL	Cell regeneration/hGH deficiency	[84]
*Chlorella ellipsoidea*	Biobalistic	Chloroplast	fGH	420 µg fGH protein/L	Growth hormone	[123]
*Dunaliella tertiolecta* and *C. reinhardtii*	Biobalistic	Plastids	Xylanase, α-galactosidas, Phytase, phosphate anhydrolase, and β-mannanase	NA	Animal feeds and biofuel production	[158]
*Haematococcus pluvialis*	Biobalistic	Chloroplast	Piscidi-4	NA	Antimicrobial	[159]

PEG: Polyethylene glycol; SKTI: Soybean Kunitz trypsin inhibitor; TSP: Total soluble protein; H5HA: Hemagglutinin-Influenza A virus; TSP: Total soluble protein, HBsAg: Hepatitis B surface antigen; HBcAgII: Angiotensin II fusion to hepatitis B virus (HBcAg); HPV16 E7: Human papillomavirus 16 E7 protein; ctxB-pfs25: Plasmodium falciparum surface protein (Pfs25) fused to cholera toxin (CtxB); pfs25 and pfs28: Plasmodium falciparum surface protein 25 and 28; c.r.pfs48/45: Plasmodium falciparum surface protein 48/45; AMA1/MSP1-GBSS: Apical major antigen or major surface protein fused to granule bound starch synthase; CTB-D2: fibronectin-binding domain D2, fused to the cholera toxin B subunit protein; hGAD65: Human glutamic acid decarboxylase; CSFV E2: classical swine fever virus structural protein E2; αCD22: Immunotoxin protein; 83K7C: Human IgG1 monoclonal antibody 83K7C against the PA83 anthrax antigen; DAB: dry algal biomass; HSV8 scfv: single-chain variable regions antibody against Herpes simplex virus glycoprotein D; HSV8-lsc: Large single-chain antibody directed against Herpes simplex virus glycoprotein D; huBuChE: A fusion protein containing luciferase and the human butyrylcholinesterase; AISD: Acquired immunodeficiency syndrome: FWB: Fresh weight biomass; IL-2 and PfCelTOS: PfCelTOS fused to human interleukin-2; sTRAIL: Tumor factor-related apoptosis inducing ligand; IFN-β1: Human interferon β1; VEGF: Human vascular endothelial growth factor; HMGB1: High mobility group protein B1; hEGF: Human epidermal growth factor; ALFPm3: Anti-Lipopolysaccharide factor isoform 3; CelK1: Bacterial endoglucanase (CelK1, Glycohydrolase, family 5) enzyme; hGH: human growth hormone, M-SAA: Bovine mammary-associated amyloid; hMT-2: Metallothionein-2; IFN-α2a: Human interferon-α; IF: Human protein intrinsic factor; WSSV VP28: White spot syndrome virus protein; ToAMP4: Taraxacum officinale antimicrobial peptide 4; hLF: Human lactoferrin; HER-2 Human Epidermal Growth Factor Receptor-2; MUC1: Mucin-like glycoprotein 1; WT1: Wilms’ Tumor Antigen; MAM-A: Mammaglobin-A; LTB:RAGE: Receptor of Advanced Glycation End products fused to *E. coli* heat-labile enterotoxin B subunit; GP1: Complex viral proteins from Zaire ebolavirus; HA: Recombinant hemagglutinin from A/Puerto Rico/8/34 (H1N1) influenza virus; fGH: flounder growth hormone.

**Table 3:** Virus-based vectors used for biopharmaceutical production.

Microalgae Host	Type of Transformation	Name	Viral Elements	Protein Expressed	References
*Schizochytrium* sp.	Transient nuclear/Inducible expression	Algevir	*Cauliflower mosaic virus*: 35S promoter 35S terminator *Ageratum enation virus:* Replication protein “Rep” Origin of replication “Ori”	The GP1 from Zaire ebolavirus and LTB RAGE (23–54 amino acids) The ZK1, ZK2, ZK3 from the E protein from Zika virus fused to LTB The multiepitope protein BCB comprised epitopes from HER-2, MUC1, WT1, MAM-A fused to LTB	[79,115,155,156]
*Chlamydomonas reinhardtii*	Transient nuclear	pBYR2e	*Cauliflower mosaic virus*: 35S promoter *Tomato bushy stunt virus:* RNA silencing suppressor P19 *Bean Yellow Dwarf Virus:* Short intergenic region SIR Long intergenic region LIR C1/C2 Replication protein and replication protein A *Tobacco mosaic virus* Ω: 5’untranslated region	The RBD from SARS-CoV-2 The bFGF	[35]
*Chlorella vulgaris*					

LTB: Bacterial toxin B subunit of the heat-labile *E. coli* enterotoxin; RAGE: Receptor of Advanced Glycation End products; ZK1: amino acids LDKQSDTQYVCKRTLVDR; ZK2: amino acids FSDLYYLTM; ZK3: amino acids LKGVSYSLCTAAFTFTKI; HER-2 Human Epidermal Growth Factor Receptor-2; MUC1: Mucin-like glycoprotein 1; WT1: Wilms’ Tumor Antigen; MAM-A: Mammaglobin-A; RBD: Receptor Binding Domain; SARS-CoV-2: Severe Acute Respiratory Syndrome Coronavirus 2; bFGF: Fibroblast Growth Factor.

**Table 4:** Molecular elements from viruses infecting microalgae tested for the expression of recombinant proteins.

Viral Genomic Element	Name	Viral Source	Size (bp)	Type of Expression	Transformation Method	Protein Expressed	Heterologous Host	Reference
Promoters	C1P1	ClorDNAV	502	Stable	Biobalistic	eGFP	Pennales diatom *Phaeodactylum tricornutum*	[181]
					Electroporation	*Sh ble*	*Chlamydomonas* reinhardtii	
	ClP2		474	Stable	Biobalistic	eGFP	Pennales diatom *Phaeodactylum tricornutum*	
	CdP1	CdebDNAV	477					
	TnP1	TnitDNAV	424					
	TnP2		424					

ClorDNAV: *Chaetoceros lorenzianus-*infecting DNA virus; CdebDNAV: *Chaetoceros debilis*-infecting DNA virus; TnitDNAV: *Thalassionema nitzschioides*-infecting DNA virus; eGFP: enhanced green fluorescence protein; *Sh ble*: bleomycin-resistant gene.

## Data Availability

Not applicable.
